# Interhemispheric asymmetry of the brain in patients with type 1 diabetes mellitus and cognitive impairment

**DOI:** 10.3389/fendo.2022.961254

**Published:** 2022-08-29

**Authors:** Yulia Gennadevna Samoilova, Mariia Vladimirovna Matveeva, Olga Sergeevna Tonkih, Dmitry Anatolievich Kudlay, Oxana Alekseevna Oleynik, Stephen Olaide Aremu, Oksana Yurievna Kilina, Alexander Federovich Kanev, Olga Mihailovna Gerget

**Affiliations:** ^1^ Department of Children Diseases, Siberian State Medical University, Tomsk, Russia; ^2^ Department of Tomographic Research Methods, Siberian State Medical University, Tomsk, Russia; ^3^ Department of Pharmacology, Ivan Mikhailovich Sechenov First Moscow State Medical University, Moscow, Russia; ^4^ Department of Internal Medicine, Katanov Khakass State University, Abakan, Republic of Khakassia, Russia; ^5^ Department of Internal Medicine with a Course of Therapy of Pediatrics Faculty, Siberian State Medical University, Tomsk, Russia; ^6^ Department of Information Technology of the Engineering School of Information Technology and Robotics, Tomsk Polytechnic University, Tomsk, Russia

**Keywords:** type 1 diabetes mellitus, interhemispheric asymmetry, brain, cognitive impairment, neuroimaging

## Abstract

With an ageing of population and a splurging epidemic of diabetes mellitus (DM), the prevalence of complications associated with pathology of the central nervous system are expected to increase, which in the future may have serious consequences for public health. It is known that one of the main manifestations of brain damage in type 1 diabetes is cognitive impairment, which is possibly associated with the peculiarities of vascularization and interhemispheric asymmetry, which requires in-depth analysis using modern neuroimaging methods. The aim of the study is to assess the symmetry of structural, metabolic and neurovascularization changes in the brain in patients with type 1 diabetes and cognitive impairment. The study included 120 patients with type 1 diabetes aged 18 to 45 years suffering from cognitive impairment, and 30 people without cognitive decline and the control group (n=30) healthy people without diabetes. Neuropsychological testing included the Montreal Cognitive Dysfunction Assessment Scale (MoCA test). For neuroimaging methods, standard magnetic resonance imaging (MRI), magnetic resonance spectroscopy (MRS), contrast and non-contrast-enhanced perfusion were used. Statistical processing was carried out using the SPSS Statistic 2020 software. In patients with type 1 diabetes with cognitive impairment, as manifested by impaired memory and/or attention, perfusion imaging revealed the presence of brain asymmetry zones. Standard MRI allowed to demonstrate changes in the white, gray matter and hippocampus in the right hemisphere. The results obtained were refined taking into account the topical localization, so during the perfusion study, regions with asymmetric blood flow were identified - namely, the white matter of the frontal lobe and the gray matter in the occipital lobe. Spectroscopy of the brain revealed that it was in these areas of the brain that the most significant metabolic disorders were noted – in the form of significantly altered ratio of N-acetylaspartate (NAA)/choline (Cho) on the left, along with the asymmetry in phosphocreatine level (Cr 2) on the right. In conclusion, early preclinical predictive diagnostics with the use of modern neuroimaging methods allows for timely detection of impaired vascularization and brain metabolism in this group of patients, However, decreased perfusion in the region within the region of frontal lobe white matter and temporal lobe grey matter, and hippocampal cell metabolism by spectra should be highlighted among the parameters Cr right and NAA/Cho left.

## Introduction

Diabetes mellitus (DM) is a metabolic disease associated with the development of acute and chronic complications (1, 2). Among the complications of diabetes, relatively less attention is paid to cognitive impairments, which are verified in some patients with type 1 and 2 diabetes. (3). Interestingly, in the early 20th century, researchers and doctors recognized that diabetic patients often complain of poor memory and lack of attention. In 1922, Miles et al. have shown, examining memory and attention, that diabetic patients performed poorly on cognitive tasks (4). The term diabetic encephalopathy was introduced in 1950 to describe complications of diabetes associated with the central nervous system (5). Other terms, such as functional disorders of the brain and central neuropathy, have also been used in the literature to describe the cognitive dysfunctions associated with diabetes (6). Modern methods make it possible to non-invasively study the morphology and functioning of the brain in diabetic patients, determining the possibilities of predictive diagnostics.

Currently, there is a sufficient number of descriptive studies of the brain in type 1 diabetes, which describe the phenomenon of focal atrophy of the white and/or gray matter, more often the frontal, temporal and occipital lobes (7, 8). Another neuroimaging method is Magnetic resonance spectroscopy (MRS) - an advanced biochemical analysis technique that detects changes in metabolic neurochemical levels of metabolites in various areas of the brain *in vivo*. In type 1 diabetes, changes in the levels of NAA, Cho and the NAA/Cr (creatine) ratio are most often recorded (9–11). Considering the peculiarity of the development of microangiopathies in patients with type 1 diabetes, the assessment of cerebral perfusion also plays an important diagnostic role, allowing for the demonstration of changes in cerebral blood flow and other characteristics of the cortical and subcortical formations of the brain (12). Therefore, the use of these methods in a complex way allows us to clarify the features of morphofunctional associations in patients with cognitive impairments. At the same time, there are data in the literature that indicate a possible connection between the asymmetry of the cerebral hemispheres and various pathological conditions, which are not caused by genetic prerequisites (13, 14).

Thus, due to the lack of systematized data on the symmetry of brain structures, we formulated the aim of the study: to assess the symmetry of structural, metabolic and neurovascularization changes in the brain in patients with type 1 diabetes mellitus and cognitive impairments.

## Methods

The study protocol was approved by the Ethics Committee of the Federal State Budgetary Educational Institution of Higher Education Siberian State Medical University of the Ministry of Health of Russia (conclusion No. 5265 of 05/02/2017). Inclusion criteria: patients with type 1 diabetes mellitus aged 18-35 years who have signed an informed consent. Exclusion criteria: other types of diabetes mellitus (type 2 or gestational diabetes mellitus), organic brain disease, psychiatric disorders, contraindications to MRI, glomerular filtration rate less than 60 ml/min, severe visual and hearing loss. The sample required for this study was calculated using IBM SPSS Sample Power software. The minimum sample size was 100 people. To increase the power of the sample size, taking into account the possibility of losing 10% of the data during the study, the sample level was 120 people. This number of participants will ensure the representativeness of the obtained sample and will allow extrapolating the obtained data to the population. A computerized randomization was used to create a list from the diabetes registry for patient recruitment.

The study included 120 patients with type 1 diabetes with cognitive impairment. The control group (n = 30) was comparable in age (26 [23:39] years) and disease duration (13 [2:24] years). The control group consisted 30 healthy volunteers selected randomly. Screening for cognitive disorders was performed using the Montreal Cognitive Assessment Scale (MoCA test). The degree of cognitive impairment was established in strict accordance with generally accepted criteria, according to the classification of Academician of the Russian Academy of Medical Sciences (15), distinguishing between severe, moderate and mild cognitive impairments.

Standard MRI examination of the brain was performed in axial, sagittal and coronal projections using T2 (TR - time of repetition) 4932 ms, TE (Echotime) 90 ms, T1 (TR 280 MS, te 6.1 MS) modes, using the programs with free water signal suppression (Fluid Attenuated Inversion Recovery, FLAIR, TR 8000 ms, TE 105 ms, TI - time in version 2200 ms) on a Signa Creator “E” magnetic resonance scanner, GE Healthcare, 1.5 T, China.

Dynamic contrast MRI was performed, using Gadovist contrast agent administered as a 5 ml intravenous bolus with acquisition of images weighted by the inhomogeneity of the magnetic field (dynamic susceptibility contrast MR), as well as the technique of arterial spin labeling (ASL), which does not require the administration of contrast agent and allows one to quantify cerebral blood flow.

To process the MRI results, the FreeSurfer program was used, which is designed to analyze and visualize the structural and functional parameters of neuroimaging from cross-sectional or longitudinal studies, which was developed by the Computational Neuroimaging Laboratory at the Center for Biomedical Imaging (http://surfer.nmr.mgh.harvard.edu/). Proton spectroscopy of the brain was performed in a multivoxel mode; in the hippocampus region, the main spectra of NAA, Cho, Cr, and Cr2, as well as their ratios, were recorded.

Statistical analysis and data processing were performed using SPSS Statistica software version 25.0 on Windows 7/XP Pro operating systems. Methods of descriptive statistics were: mean value and standard deviation - for normally distributed data; quartiles - for non-normally distributed data. Qualitative binary signs were presented in the form of relative frequency (%) and its 95% confidence interval. Testing of statistical hypotheses of normally distributed quantitative parameters was performed using the following parametric criteria: Student’s t - test for paired comparison (when assessing independent samples), Student’s t - test for dependent data (dependent), analysis of variance for multiple comparisons. Correlation analysis was assessed using Pearson’s criterion. Nonparametric criteria were used to test hypotheses for non-normally distributed quantitative parameters: Mann-Whitney (independent samples) and Wilcoxon (dependent samples), Kruskal-Wallace criteria for paired comparison. For correlation analysis - nonparametric Spearman criterion. To assess reliability of differences in qualitative characteristics, we used conjugation tables with the calculation of χ² (chi-square). The null hypothesis was rejected at the level of statistical significance p < 0.05. 

## Results

According to the study, in patients with type 1 diabetes, cognitive impairment was presented as mild in 50.8% (n = 61) of the patients, moderate in 40% (n = 48) and severe in 9.2% (n = 11). Neuropsychological testing data showed a decrease in the overall score of the MoCA test and lower scores on the tasks for attention (serial subtraction) and memory (p <0.001).

The characteristics of the patients are presented in **Table 1**, the groups were comparable, with the exception of the level of fasting glycemia.

**Table 1:** Characteristics of patients with type 1 DM (Me [Q1; Q3]).

Parameters	Patients with type 1 DM and cognitive impairment, n = 120	Patients with type 1 DM without cognitive impairment, n = 30	Patients without DM, n = 30	*P*
Age, years	27[18:45]	26 [23:39]	27 [23:39]	0,2
Disease duration, years	11 [1:32]	13 [2:24]	–	0,2
Fasting plasma glucose, mmol/l	9.1 [6.4:16.4]	7,9 [5.5:18.3]	4,9 [3.8:5.3]	0,05
HbA1c,%	7.6 [6:12.4]	6.9 [4.5:10.3]	4.9 [3.8:5.8]	0,2
Body weight index, kg/m^2^	22.6 [17.4:30.6]	21.8 [16:30.4]	21.7 [18:29.3]	0,2

p ≤ 0,05 – significant differences.

Next, we assessed the presence of microvascular complications in patients in both groups (**Table 2**). Differences in the incidence of retinopathy and nephropathy between groups were found.

**Table 2:** Characteristics of patient groups according to the presence of microvascular complications of type 1 DM.

Complications	Patients with type 1 DM and cognitive impairment, n = 120	Patients with type 1 DM without cognitive impairment, n = 30	*Р*
Angioretinopathy,%	75%	63,3%	0,045
Polyneuropathy,%	62,4%	70%	0,359
Nephropathy, %	45,8	20%	0,025

p ≤ 0,05 – significant differences.

Anamnesis analysis of patients with type 1 diabetes revealed the following concomitant thyroid diseases: autoimmune thyroiditis, nodular goiter in compensation stage - 6 (5%) cases in patients with cognitive impairment and 2 (6,6%) without, allergic reactions to food or medications - 12 (10%) and in 3 (10%) patients, gastrointestinal diseases (gallstone disease) and kidney diseases (chronic pyelonephritis) - 2 cases (1.6%) and in 1 (3,3%).

Patients with cognitive impairment were more likely to be on multiple insulin injections - 90 (75%), when in the group without cognitive impairment most received insulin on a pump regimen -24 (80%).

Factor analysis revealed that patients with type 1 diabetes and cognitive impairment were more often on artificial feeding 30% (n=36), regularly consumed coffee 85%(n=102) and alcohol 58,3% (n=70), smoked a third of all patients 40% (n=48), and half had higher education 50% (n=60) and in more than half of the cases had a full family 78,3% (n=94). Whereas patients with type 1 diabetes and without cognitive impairment were naturally feeding 73,3% (n=22), frequently consumed coffee 93,3% (n=28), and less than half smoked 40% (n=12), alcohol was consumed by 20% of the subjects (n=6), almost all of the patients had a college education 86,6% (n=26) and most had full families 83,3% (n=25).

### Interhemispheric asymmetry according to standard brain MRI

Initially, the method of segmentation was used to assess the volume of brain structures, as a result of which the total volumes of white, gray matter, including the hemisphere and hippocampus, were obtained (**Table 3**).

**Table 3:** Brain segmentation in patients with type 1 DM.

Anatomic region	Patients with type 1 DM, n = 150		Patients without DM, n = 30	*P*
	With cognitive impairment, n = 120	Without cognitive impairment, n = 30		
Grey matter, mm^3^	478009 [457669,1-511273,8]	497704 [442993,1-586559,6]	626496 [593716; 649388]	0,106
Grey matter, left hemisphere, mm^3^	225046 [212392,9-232197,2]	252441 [224292,9-271860,4]	295734 [278564; 301837]	0,0004
Gray matter, right hemisphere, mm^3^	235085 [219200,2-254713,2]	253587 [223200,3-270387,4]	298546 [278058;320759]	0,015
White matter, mm^3^	455968 [421138,4- 473940,5]	503517 [440036,6- 509720,7]	659724 [6391765;689266]	0,005
White matter, left hemisphere, mm^3^	232213 [217884,9-239910,2]	241831 [217516,8-264238,8]	359624 [338616;378166]	0,639
White matter, right hemisphere, mm^3^	235509 [211609,8-237965,5]	270207 [228287,6-310513,8]	365926 [329764;387165]	0,046
Left hippocampus, mm^3^	73 [72,1-73,4]	73 [72,9-74,8]	77,8 [76,8;79,2]	0,141
Right hippocampus, mm^3^	72 [71,1-73,2]	73 [72,4-75,0]	78,3 [76,3;79]	0,005

p ≤ 0,05 – significant differences.

In patients with type 1 diabetes and cognitive impairments, changes were noted in the volumes of white matter of both hemispheres, gray matter and the hippocampus on the right, compared to the patients from the control group, which indicate signs of atrophy in aforementioned areas.

### Interhemispheric asymmetry according to contrast and non-contrast cerebral perfusion

We defined the null hypothesis as the absence of significant differences between the perfusion indices of the right and left cerebral hemispheres, while the alternative hypothesis is the presence of significant differences between the samples. To accept or reject this paradigm, the Mann-Whitney test was applied. **Table 3** shows the values ​​of the Mann-Whitney U-statistics for which the alternative hypothesis is accepted, that is, statistically significant differences between the samples are proved.

**Figure 1:**
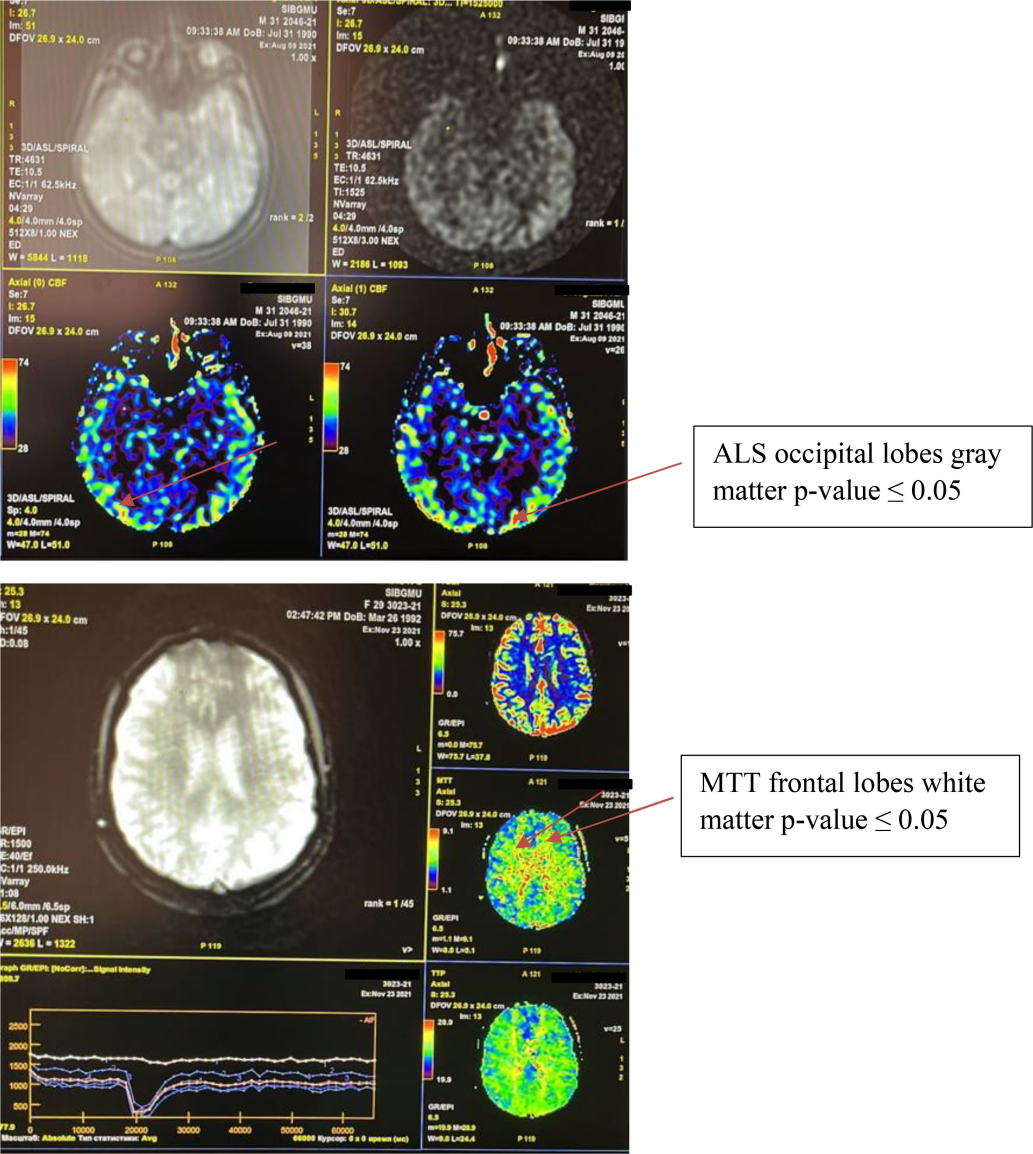
An image depicting an example of asymmetry perfusion in patent with diabetes type 1.

Thus, during contrast perfusion in the area of the white matter of the frontal lobe, asymmetry was observed in the parameter of the average time of blood passage. When assessing non-contrast perfusion, differences were shown in the main parameter of cerebral blood flow in the occipital lobe of the gray matter in patients with type 1 diabetes and cognitive impairment. Apparently, this is an example of how the processes of neuroplasticity are implemented, aimed at compensating for cognitive impairments (**Figure 1**).

Cerebral perfusion was lower in the group with moderate cognitive impairment than in patients with mild (p ≤ 0,009), whereas no significant differences were recorded in the temporal lobe region (**Table 4**).

**Table 4:** Asymmetry of neurovascularization according to contrast and non-contrast perfusion in patients with type 1 diabetes mellitus and cognitive impairment.

Localization and indices of perfusion	U-statistics	P value
Frontal lobe, white matter, MTT/sec	201,5000	0,032032
Temporal lobe, grey matter, ASL/CBF	201,5000	0,032032

U-Mann-Whitney test, p-value ≤ 0.05; MTT, mean transit time; AS, arterial spin labeling - method of spin labeling of arterial blood; CBF, cerebral blood flow.

### Interhemispheric asymmetry as assessed using proton spectroscopy of the brain

Based on the initial data of proton spectroscopy, a correlation matrix was built to check the presence of variables that have a high degree of connection with each other during proton spectroscopy of the brain (**Table 5**).

**Table 5:** Correlation matrix for checking the normality of parameters of proton spectroscopy of the brain.

	NAA left	NAA right	Cho left	Cho right	Cr left	Cr right	Cr2 left	Cr2 right	NAA/Cr left	NAA/Cr right	NAA/Cho left	NAA/Cho right	Cho/Cr left	Cho/Cr right
NAA left	1	0,5	0,01	-0,9	0,2	0,3	0,3	0,4	-0,1	0,02	-0,5	-0,2	-0,1	-0,4
NAA right	0,5	1	0,22	0,2	0,3	0,3	0,6	0,4	0,1	0,2	-0,3	-0,4	-0,0	-0,4
Cho left	0,01	0,22	1	0,4	0,2	0,4	0,1	0,1	0,7	0,7	0,4	0,2	0,6	-0,2
Cho right	-0,9	0,2	0,4	1	-0	-0	0,1	-0,1	0.1	0,1	0	0,1	0,45	0,4
Cr left	0,2	0,3	0,2	-0	1	0,3	0,1	0,2	0,3	0,1	-0	0	-0,3	-2,3
Cr right	0,3	0,3	0,4	-0	0,3	1	0,1	0,4	0,6	0,6	0,1	0,2	0,1	-0,5
Cr2 left	0,3	0,6	0,1	0,1	0,1	0,1	1	0,5	-0,1	0	-0,4	-0,4	0	-0,3
Cr2 right	0,4	0,4	0,1	-0,1	0,2	0,4	0,5	1	0,16	0,15	-0,1	-0,1	0	-0,3
NAA/Cr left	-0,1	0,1	0,7	0,1	0,3	0,6	-0,1	0,16	1	0,8	0,65	0,4	0,4	-0,2
NAA/Cr right	0,02	0,2	0,7	0,3	0,1	0,6	0	0,15	0,8	1	0,35	0,47	0,6	-0,3
NAA/Cho left	-0,5	-0,3	0,4	0	-0	0,1	-0,4	-0,1	0,65	0,35	1	0,5	0,2	0,3
NAA/Cho right	-0,2	-0,4	0,2	0,1	0	0,2	-0,4	-0,1	0,4	0,47	0,5	1	0,2	0,4
Cho/Cr left	-0,1	-0,0	0,6	0,45	-0,3	0,1	0	0	0,4	0,6	0,2	0,2	1	0,1
Cho/Cr right	-0,4	-0,4	-0,2	0,4	-2,3	-0,5	-0,3	-0,3	-0,2	-0,3	0,3	0,4	0,1	1

NAA, N-acetylaspartate; Cho, choline; Cr, creatine; Cr2, phosphocreatine.

The figure shows that the relationship between the variables exists, but it is not pronounced enough to exclude any variable, since the maximum coefficient does not exceed 0.8. Therefore, based on the correlation matrix, no feature can be rejected as uninformative. The Kruskal-Wallis test was used to check the informativeness of the features (**Table 6**).

**Table 6:** Values of the Kruskal-Wallis rank test for parameters of proton spectroscopy of the brain of patients with type 1 diabetes mellitus.

Parameter	Statistics	p-value
**NAA left**	22,62228	0,0001
**NAA right**	26,53589	0,0003
**Cho left**	28,52084	0,0000
**Cho right**	14,18308	0,0027
**Cr left**	5,721290	0,1260
**Cr right**	33,81858	0,0000
**Cr2 left**	29,96852	0,0002
**Cr2 right**	34,03594	0,0001
**NAA/Cr left**	33,19992	0,0000
**Naa/Cr right**	31,45471	0,0001
**NAA/Cho left**	34,10368	0,0004
**Naa/Cho right**	33,72973	0,0001
**Cho/Cr left**	23,58656	0,0000
**Cho/Cr right**	18,70439	0,0003

NAA, N-acetylaspartate; Cho, choline; Cr, creatine; Cr2, phosphocreatine.

**Figure 2:**
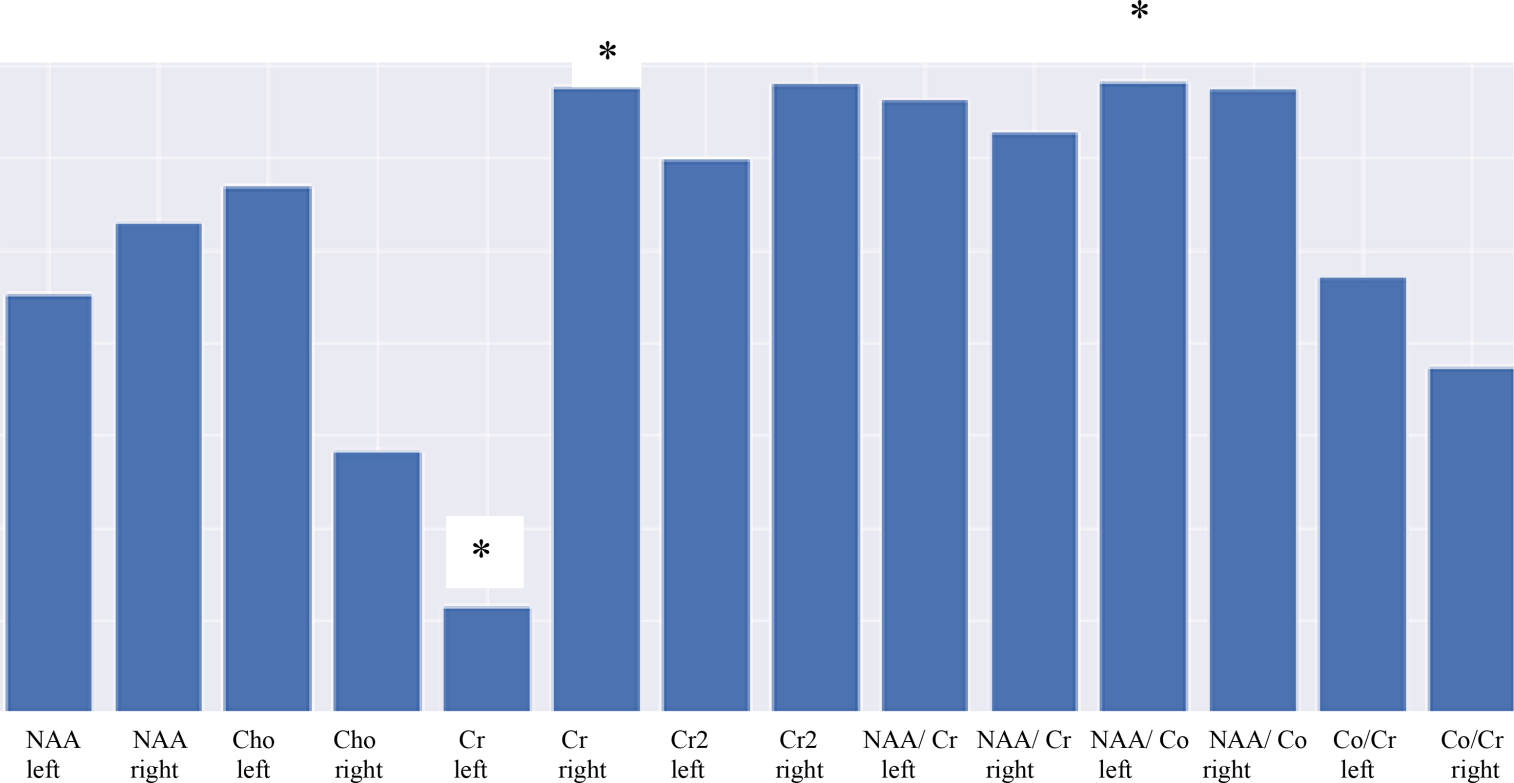
Diagram showing the significance of MRS parameters highlighting the importance of features. * - statistical significance (p ≤ 0,05).

Based on the data in the table, we can conclude that for each variable the hypothesis of significance does not change at the significance level of 0.5, because the p-value does not exceed this figure. **Figure 2** shows a diagram of the distribution of the importance of features

**Figure 2** demonstrates that the least informative trait is Cr on the left, with the most informative being NAA/Cho on the left and Cr2 on the right. Thus, the asymmetry of the hippocampal region on the right is associated with a change in metabolism depicted as the change in the NAA/Cho ratio.

An analysis was performed during the study, which showed the following clinical features in patients who had interhemispheric asymmetry by all MRI methods of investigation: duration of disease more than 5 years, HbA1c more than 7.5% (and time in the target range less than 65%) and those who were on multiple insulin injections, by neuropsychological tests they usually had 2 domains of cognitive functions affected-attention and another of those presented (memory, visual-construction skills, speech fluency). These data confirm the importance of distinguishing risk groups of patients for the development of cognitive impairment and morpho-functional changes of the brain in diabetes mellitus especially at a young age.

## Discussion

As a result of the study, it was revealed that type 1 diabetes mellitus is characterized by mild to moderate cognitive impairment with a predominance of impaired attention and memory. It should be noted that memory impairment in this type of diabetes has not been previously recorded, which may require the use of specific tests to understand the phenomenology of this result. These data contradict the results of many authors, who believe that with type 1 diabetes, the neurodynamic component of cognitive functions is mainly affected, namely the pace and the ability to concentrate (16), although there are studies that focus on memory impairment primarily associated with diabetes (17, 18).

For example, a study conducted by Weinger etal. (19) using magnetic resonance imaging as the diagnostic tool and with a focus on the cerebral white mater revealed that the participants who had type 1 diabetes had comparatively lower scores to the controls on one measure of executive function (Sorting Test), short-term memory, delayed recall, vocabulary, and psychomotor efficiency.

With regard to the issues of asymmetry of the cerebral hemispheres, many studies are devoted to the issues of sensorimotor integration (20, 21). On the other hand, the different functioning of the cerebral hemispheres is an important phenomenon in injury, employing the rehabilitation potential and neuroplasticity of the central nervous system (22). The only large meta-analysis in neurology on hemispheric asymmetry was presented in 2019, consisting of 159 publications on voxel-based morphometry (registration of 4469 patients and 4307 controls), showing that asymmetry does exist in neurodegenerative diseases. Regions with asymmetric brain decline were located in areas primarily affected by neurodegeneration. Thus, with moderate cognitive impairment, the region of the right hippocampus is most vulnerable (23).

The data obtained in this study deserve attention from the perspective of preventive medicine, since early preclinical diagnosis of cognitive impairment and related dysfunctions, including microangiopathies, can reduce health care costs and improve the quality of life of patients with type 1 diabetes. Information on non-invasive techniques seems promising: non-contrast perfusion, in which the area of ​​interest is the gray matter of the occipital lobes; and proton spectroscopy of the hippocampus, the informative signs for which are the NAA/Cho ratio on the left and the Cr2 content on the right.

The study isn’t without limitations. For instance, the associations used in this type of study does not allow for the assessments of the direction of causality, however the long duration diabetes in the participants makes causation in the reverse direction less probable. Furthermore, the study has relatively low power given the limited sample size; hence, an expansion of the patient sample is required in further studies. It’s also important that longitudinal studies are planned to confirm the findings of the study and to determine whether they can predict cognitive impairment and associated neuroimaging changes as well as related dysfunctions, including microangiopathies, in type 1 diabetes mellitus. The strengths of the study include the study sample source, the holistic nature of the cognitive assessment that included clinical and neuropsychological assessments and the neuroimaging methodology that comprise of updated, advanced, automated volume measures that gave precise and accurate measures and assessments (24)

## Conclusion

In patients with type 1 diabetes with a disease duration of more than 10 years, the neurodynamic type of cognitive impairment can turn into cortical-subcortical one, taking into account the topical localization of the revealed changes. Asymmetry of the hemispheres is characteristic of patients with type 1 diabetes and cognitive impairment. Early preclinical predictive diagnostics with the use of modern neuroimaging methods allows for timely detection of impaired vascularization and brain metabolism in this group of patients.

Thus, asymmetry according to various methods may be key in the predictive diagnosis of cognitive impairment in type 1 diabetes mellitus. Asymmetry in the volume of gray, white matter of the brain and hippocampus, decreased perfusion in the region within the region of frontal lobe white matter and temporal lobe grey matter, and hippocampal cell metabolism by spectra should be highlighted among the parameters Cr right and NAA/Cho left.

However, further work is needed to validate the findings and provide a better understanding of the functional role of interhemispheric asymmetry, for example, in the context of cognitive reserve and compensation.

## Data availability statement

The raw data supporting the conclusions of this article will be made available by the authors, without undue reservation.

## Ethics statement

The studies involving human participants were reviewed and approved by The study protocol was approved by the Ethics Committee of the Federal State Budgetary Educational Institution of Higher Education Siberian State Medical University of the Ministry of Health of Russia (conclusion No. 5265 of 05/02/2017). The patients/participants provided their written informed consent to participate in this study.

## Author contributions

All authors listed have made a substantial, direct, and intellectual contribution to the work and approved it for publication.

## Funding

With the support of a grant from the President, agreement 075-15-2020-192 on 19.03.2020; No 075-15-2022-599 on 06.05.2022.

## Conflict of interest

The authors declare that the research was conducted in the absence of any commercial or financial relationships that could be construed as a potential conflict of interest.

## Publisher’s note

All claims expressed in this article are solely those of the authors and do not necessarily represent those of their affiliated organizations, or those of the publisher, the editors and the reviewers. Any product that may be evaluated in this article, or claim that may be made by its manufacturer, is not guaranteed or endorsed by the publisher.
